# An artificial intelligence-assisted digital microfluidic system for multistate droplet control

**DOI:** 10.1038/s41378-024-00775-5

**Published:** 2024-09-27

**Authors:** Kunlun Guo, Zerui Song, Jiale Zhou, Bin Shen, Bingyong Yan, Zhen Gu, Huifeng Wang

**Affiliations:** Key Laboratory of Smart Manufacturing in Energy Chemical Process Ministry of Education, East China University of Science and Technology, Shanghai, China

**Keywords:** Electrical and electronic engineering, Chemistry

## Abstract

Digital microfluidics (DMF) is a versatile technique for parallel and field-programmable control of individual droplets. Given the high level of variability in droplet manipulation, it is essential to establish self-adaptive and intelligent control methods for DMF systems that are informed by the transient state of droplets and their interactions. However, most related studies focus on droplet localization and shape recognition. In this study, we develop the AI-assisted DMF framework μDropAI for multistate droplet control on the basis of droplet morphology. The semantic segmentation model is integrated into our custom-designed DMF system to recognize the droplet states and their interactions for feedback control with a state machine. The proposed model has strong flexibility and can recognize droplets of different colors and shapes with an error rate of less than 0.63%; it enables control of droplets without user intervention. The coefficient of variation (CV) of the volumes of split droplets can be limited to 2.74%, which is lower than the CV of traditional dispensed droplets, contributing to an improvement in the precision of volume control for droplet splitting. The proposed system inspires the development of semantic-driven DMF systems that can interface with multimodal large language models (MLLMs) for fully automatic control.

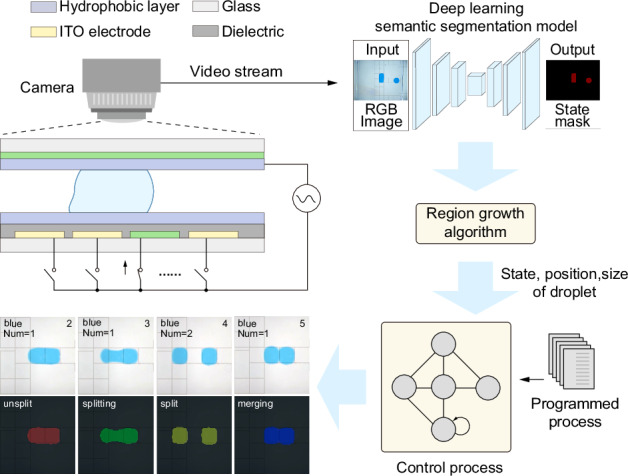

## Introduction

Digital microfluidics (DMF) has become a prevalent technique for handling liquids because of its unique flexible and discrete fluid control capabilities^1–3^. In particular, DMF systems based on electrowetting-on-dielectric (EWOD) principles utilize voltage modulation to control the wettability of liquids on solid surfaces, facilitating the manipulation of droplets across scales from nL to μL^4–7^. This technique has advantages such as miniaturization, programmability, parallelization, and low power consumption^8,9^. Owing to these benefits, DMF devices have been used in a wide range of applications, including point-of-care testing^10,11^, cell research^12^, biomedicine^13,14^, and environmental monitoring^15^.

Typically, DMF devices accept a standard set of basic instructions to perform droplet manipulations. Users can create droplet actuation sequences for each actuating electrode^16,17^, thereby allowing the droplets to move, dispense, merge, split, and mix^18–20^. Typically, an automated DMF system incorporates sensors based on imaging or impedance detection to monitor droplet operations in real time^21–24^. The control algorithm, including droplet tracking and path planning on the basis of feedback signals^25,26^, is also integrated into the DMF system to manage electrode switching and sequencing for continuous droplet manipulation. These sensing and control methods not only enhance the droplet manipulation stability but also optimize the motion performance of the droplet^25^. Image processing methods, which include background subtraction methods, edge detection, object detection, and image segmentation^27–30^, are applied to droplet motion recognition; they offer a noncontact, real-time solution for signal acquisition, resulting in rapid and accurate detection and tracking of droplets, including transparent droplets^31^. In practice, the operations of droplets often result in changes in droplet appearance, such as changes in shape and color. However, few studies have focused on the automated control of droplets with recognition of their states on the basis of appearance changes.

In the past decade, the advancement of convolutional neural networks has led to the birth of the first high-impact segmentation models, trained end-to-end, pixel-to-pixel, which exceed the state-of-the-art in semantic segmentation^32^. Next, encoder‒decoder segmentation models, such as DeeplabV3+ and U-Net, were proposed to improve segmentation-intensive reading by mapping low-resolution encoder features to full-input resolution feature maps^33^. Compared with image processing methods based on edge detection and object detection^34^, semantic segmentation techniques stand out for their outstanding advantages in complex scenarios and multicategory object segmentation tasks, including precise object segmentation, support for multiple object categories or states, environmental awareness, and advanced image analysis applications^35–37^. Therefore, they have been widely applied in AI-assisted technology, such as autonomous driving, robotics, and smart health care^38–40^. Considering the transparency, uncertain boundaries, and high degrees of freedom of droplet operations in DMF, semantic segmentation techniques can be used to determine the edges and sizes of droplets accurately and provide precise recognition of multistate droplets in DMF. Coupled with control methods, semantic segmentation facilitates the development of fully automated and flexible control systems to execute complex tasks. However, there are still challenges for the implementation of semantic segmentation-based DMF control, including establishing a dataset for training the deep learning model, converting the image data into droplet states in a dynamic manner and translating the state-related control process into the voltage state of the actuating electrodes.

**Fig. 1:**
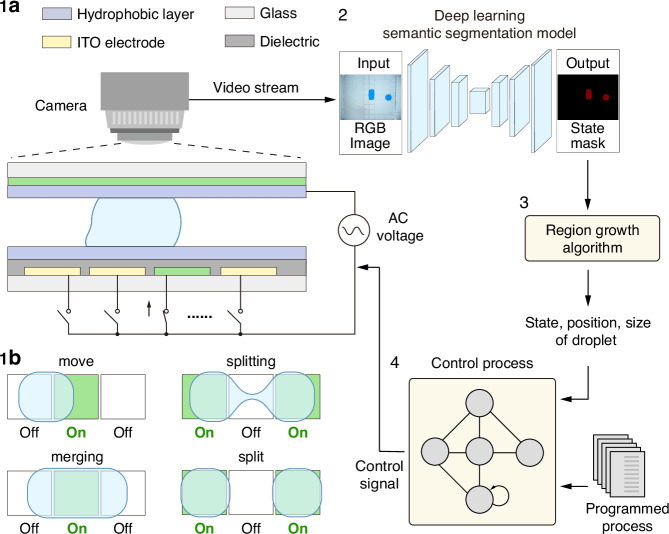
Overview of the artificial intelligence-assisted digital microfluidics system μDropAI. From left to right, top to bottom, in sequence: (1a) a hardware system for droplet actuation and video capture; (1b) manipulation of droplets; (2) a semantic segmentation model trained to recognize the droplet states at the pixel level; (3) a region growth algorithm to extract the position and morphological information of the droplets; and (4) automated control processes for user-programmed automatic control

In this work, we develop the artificial intelligence-assisted digital microfluidics system μDropAI as an implementation of the semantic segmentation model-based DMF system for highly automated multistate droplet control (Fig. 1). The framework contains four parts: (1) a hardware system for droplet actuation and video capture; (2) a semantic segmentation model trained to recognize the droplet states; (3) a region growth algorithm to extract the position and morphological information of droplets; and (4) automated control processes for user-programmed automatic control. An open-source dataset is established with labeled droplet images consisting of different states, colors and shapes. The proposed DMF system integrates the semantic segmentation model trained on the dataset to recognize droplet multistates, controls the activation sequence of electrodes, and is successfully used for automatic droplet splitting, movement, and dispensing. To the best of our knowledge, this is the first study to use semantic segmentation for the control of DMF systems. Both the dataset and μDropAI are open source. In addition, the proposed control system is compatible with existing DMF devices integrated with digital cameras such as DropBot, openDrop, and DropLab^16,41,42^; therefore, it can be widely used in DMF-based applications and promotes the integration of more AI-based techniques to be utilized in DMFs such as reinforcement learning and ChatGPT^43^.

## Methodology

### Encoder-decoder semantic segmentation model

**Fig. 2:**
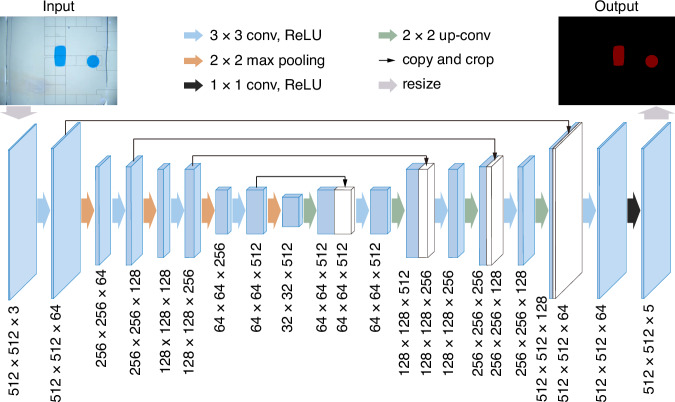
Overall structure of our model. (1) Input 1920 × 1080 images of droplets in different states; (2) downsample the image pixel and channel count (512 × 512 → 256 × 256, 64 → 5) and upsample the image pixel and channel count (256 × 256 → 512 × 512, 3 → 64); and (3) output 1920 × 1080 labeled images

A deep learning-based algorithm that automatically segments droplet multistates is proposed. Based on the traditional U-Net^44^, the proposed model is optimized for processing the images of the droplets in the DMF system with an encoder-decoder structure. The encoder unit of the proposed model performs feature extraction, and the decoder unit performs upsampling operations. Shortcut connections connect the corresponding feature maps between the encoder and decoder units (Fig. 2 and Supplementary Table S1). Incorporating these connections, the model can capture both high-level and low-level features.

Since the images acquired during DMF control contain only droplets with clear backgrounds, there are stacked convolutional blocks designed to extract features in the encoding stage. In the shallow layers, when fewer convolutional layers (2 layers) are used, the model rapidly captures the essential features of the droplets, such as edges and shapes. In the deeper layers, the use of multiple convolutional layers (3 layers) enables the extraction of more complex semantic information about the droplet, such as state and position. Within each convolution block, there are convolution layers (Conv2d) that use 3 × 3 convolution kernels with a stride of 1. These convolution layers are followed by ReLU activation and a 2 × 2 maximum pooling layer, which improves the feature expression ability of the model and reduces the spatial size of the feature maps. Compared with the original U-Net encoders, consecutive convolutional and pooling operations are utilized to reduce the parameter count. Additionally, deep stacks of convolutional layers can also learn more intricate feature representations.

Considering the high-resolution imaging of the hardware system, a direct twofold upsampling approach is introduced in the decoding stage. This structure quickly restores the original resolution and simplifies the traditional step-by-step upsampling process. The 8 convolutional layers are set to match the same resolution of the encoder. The skip connections are utilized to connect the feature maps from the encoder and decoder stages. These approaches retain more spatial information and detail, thus assisting the network in obtaining more accurate segmentation results. Compared with the U-Net decoder, direct twofold upsampling reduces computational and memory costs, making the model more lightweight.

The final layer of the network is a 1 × 1 convolutional layer with 5 channels. This layer maps the feature maps from the decoder to the same number of channels as the number of label categories considered in this paper. This approach ensures that the predicted image segmentation results are accurate.

### Region growing algorithm

The region growing algorithm (Supplementary Information S1 and Supplementary Fig. S1) is introduced to obtain a pixel-level division of the droplet state in each frame. Additionally, these segments can be connected on the basis of pixel similarity to obtain a more accurate representation of the droplet. This algorithm has a strong ability to segment and compute regions on the basis of pixel similarity^45^.

### Automated control process based on droplet states

**Fig. 3:**
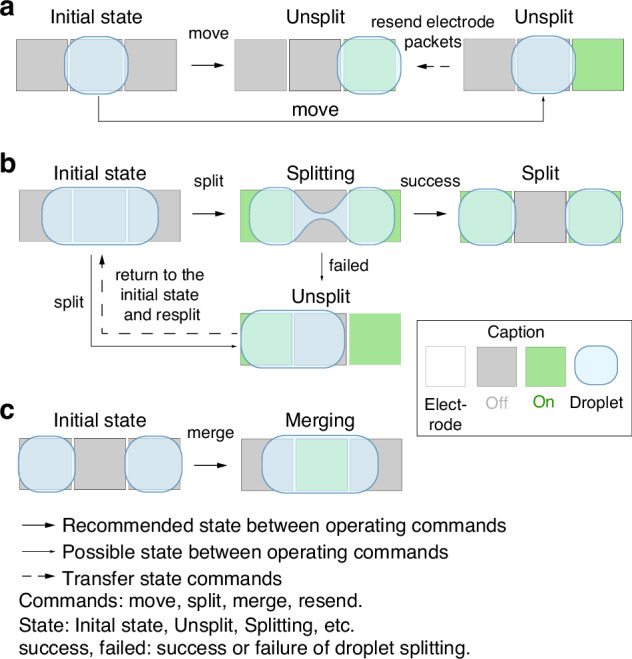
Schematic diagram of the droplet states in the control processes. **a** Droplet movement control process based on states; **b** droplet splitting and movement control process based on states; **c** droplet merging control process based on states

The control process is executed using a state machine implementation, enabling automated manipulations, such as droplet movement, splitting and merging, following user-provided manipulation instructions (Fig. 3a–c). This process begins with the translation of user commands into specific electrode-switching sequences. These sequences dictate the activation and deactivation of electrodes to control the manipulations of droplets on the chip. After initiating electrode control, a predefined delay is incorporated to allow the droplet to achieve the desired manipulations. The camera captures images of each droplet, and these images are analyzed by the semantic segmentation model to determine each droplet’s semantic information, such as state and position. Based on the analysis results, a feedback control method dynamically adjusts the electrode switching sequence to correct the incorrect operation of the droplet (Supplementary Information S5).

## Experimental methods

### Fabrication of the DMF chip

The indium tin oxide (ITO)-based DMF chip is assembled from a bottom plate (52 × 50 mm, 1.1 mm thick, ~8 Ω/sq) and an upper plate (20 × 40 mm, 0.7 mm thick, ~8 Ω/sq). A UV laser (355 nm, 5 W) with a pulse duration of 20 μs is used to process the electrode pattern on the bottom ITO plate^46^. After pattering, the ITO glass (Luoyang Guluo Glass, China) is washed with IPA and ultrapure water for 15 min each to remove the residuals and then dried with nitrogen. Before Parylene C coating, air plasma treatment is used to hydroxylate the surface of the bottom ITO plate for 2 min, and the pad area for the external connection of the bottom plate is covered with masking tape. The bottom plate is then coated with approximately 3 μm of Parylene C as the dielectric layer. Both plates are spin-coated with CYTOP as a hydrophobic layer. The masking tape on the bottom plate is then removed carefully, and the two plates are assembled using double-sided tape with a thickness of approximately 0.5 mm.

### Setup of the DMF hardware

A homemade DMF system is used to manipulate the DMF chip and control the droplet manipulation processes. The optocoupler switch array used for controlling the high-voltage source (ATG-2081, Aigtek, China) applied to the electrodes on the chip is configured by a microcontroller unit (STM32L432, ST, Italy), which receives instructions from a Python script embedded with the proposed AI-assisted control model. A microscope equipped with a CCD camera (IUA20000KPA, ToupTek, China) is positioned above the DMF chip to observe and capture images of droplet manipulation. The resolution of the CCD camera is set to 2560 × 1440 to obtain clear images. The images are subsequently transmitted to a computer with an R7 5700X CPU, 16 GB of RAM, and an RTX 3070 GPU for further processing and analysis.

### Dataset establishment

A multistate droplet dataset is established with a digital camera. Images of various droplet manipulations are captured at a frame rate of 30 frames per second. The captured videos are converted into a series of images (1920 × 1080) with OpenCV. These droplet manipulation images are labeled according to four types of states: “unsplit”, “splitting”, “split”, or “merging” with LabelMe (Supplementary Table S2) and converted to the PASCAL VOC data format. The dataset is divided into training and validation sets at a ratio of 0.8:0.2, including 3503 images in the training set and 876 images in the validation set. The training set is used to train the model, and the validation set is used to evaluate the model’s performance. The dataset is in the public domain for others (https://github.com/Eric1105/-DropAI). This protocol can be used as a reference for establishing other DMF droplet manipulation datasets.

### Model training parameters and evaluation metrics

The training GPUs were dual RTX3090 12 G. The experiment was conducted using the PyTorch framework with the programming language Python version 3.8 on a Windows system.

To adapt to the dataset, which has a large proportion of background and a small proportion of objects (Supplementary Fig. S9b), the following optimized parameter is set to accelerate the model’s convergence. After our testing, Adam was chosen as the optimizer because it has the ability to quickly identify the optimal parameter configuration to achieve better training performance. The learning rate was set to 0.0001, with a minimum learning rate of 0.000001. The learning rate is reduced every 5 epochs via the cosine annealing learning rate scheduling strategy. This approach helps gradually lower the learning rate, which allows the model to converge more smoothly and avoid excessive oscillations during training.

Since the images acquired during DMF control contain only droplets with a large proportion of background (Supplementary Fig. S9b), there is a significant imbalance of positive and negative samples in the DMF. Therefore, Dice loss is employed as the loss function (Eq. 1). A lower value of Dice loss indicates greater similarity between the predicted and ground truth results.

1 $$loss\,=\,1-2\,\times \frac{|x\cap y|}{|x|+|y|}$$

where $x$ represents the predicted segmentation mask and $y$ represents the ground truth segmentation mask.

The mean intersection over union (mIoU) is employed as the evaluation indicator for the semantic segmentation performance (Eq. 2). This metric is the average of the ratio of the intersection and union of various real labels and prediction results as follows:

2 $$mIoU\,=\,\frac{1}{n+1}\mathop{\sum }\limits_{i=0}^{n}\frac{{p}_{ii}}{{\sum }_{j=0}^{n}{p}_{ij}+{\sum }_{j=0}^{n}{p}_{ji}-{p}_{ii}}$$

where $n$ represents the total number of categories; ${p}_{ii}$ represents the number of correct predictions; ${p}_{ij}$ represents false negatives, in which $i$ is predicted as $j$; and ${p}_{ji}$ represents false positives.

## Results and discussion

### Semantic segmentation model of the droplet state

To facilitate the automatic droplet control process, the droplet states are defined as “unsplit”, “splitting”, “split” or “merging”. The unsplit state is a single droplet without any interaction with other droplets. The splitting state is a droplet with an hourglass shape. The split state involves two droplets that are separated from each other at a distance equal to the width of an electrode. The merging state involves two droplets just touching each other. To validate the segmentation performance of the proposed model on our dataset, the model’s segmentation results, mIoU, and loss are used to analyze performance. The mIoU indicates the extent to which the model-predicted area overlaps with the actual area. The loss is used to measure the difference between the model prediction and the actual target.

**Fig. 4:**
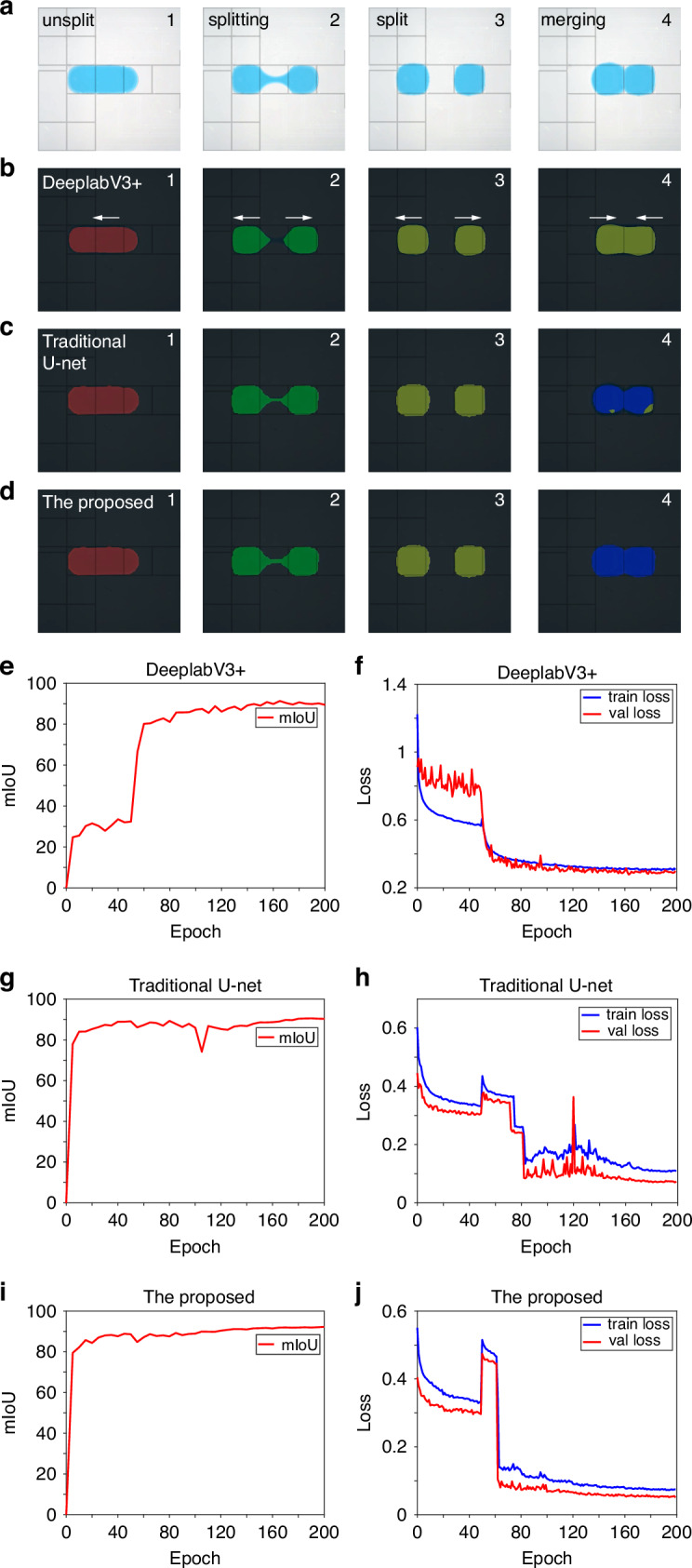
Training and evaluation of the semantic segmentation model. **a** Original images; **b** segmentation results using DeeplabV3+; **c** segmentation results using the traditional U-Net; **d** segmentation results using the proposed method; **e** mIoU of DeeplabV3+ (The mIoU indicates the extent to which the model-predicted area overlaps with the actual area.); **f** loss of DeeplabV3+ (The loss indicates the difference between the model prediction and the actual target.); **g** mIoU of the traditional U-Net; **h** loss of the traditional U-Net; **i** mIoU of the proposed model; **j** loss of the proposed model

Figure 4a–d illustrates the process of droplet splitting. As observed, the droplet transitions from the initial unsplit state (red mask) to the splitting state (green mask), culminating in the yellow split state (yellow mask). In a comparative experiment, droplets in the unsplit and split states are accurately recognized using DeeplabV3 + ^47^, traditional U-Net^44^, and our proposed model (Fig. 4b1–d1, b3–d3). During the splitting process, an hourglass shape forms inside the droplet, which indicates whether the two parts of the droplet have begun to split. In our dataset, this state is labeled the splitting state. This state was not correctly segmented by DeeplabV3+ (Fig. 4b2), as the neck of the hourglass shape was missed. In contrast, the splitting droplet is successfully segmented by both U-Net and our proposed model (Fig. 4c2, d2). Accurate recognition of the splitting state is crucial for a successful splitting operation. Once two droplets touch, the merging and split states are reliably distinguished by the proposed model (Fig. 4d4). In contrast, merging is incorrectly recognized as split by DeeplabV3+ (Fig. 4b4), and merging droplets are recognized as split by U-Net (Fig. 4c4). This difference can be attributed to the traditional U-Net using two convolutional layers in each convolution block, thereby capturing less semantic information about the droplet. DeeplabV3+ combines atrous convolution for feature extraction, thereby missing more details, including edges and semantic information about the droplet. The proposed model uses fewer convolution layers for shallow feature extraction and more convolution layers for deep feature extraction; it also applies twofold upsampling in the decoding stage, enhancing the performance for droplet segmentation and reducing the computational complexity.

The mIoU of the validation set for the proposed model is 89.73%, which is greater than the mIoU for the DeeplabV3+ (82.53%) and traditional U-Net models (84.69%) (Table 1). A higher mIoU indicates better segmentation performance, with the proposed predicted regions overlapping more closely with the actual regions. Moreover, the loss of the validation set for the proposed method stabilizes at 0.11, which is 50% less than the loss of DeeplabV3+ (0.22) and 21% less than the loss of U-Net (0.14) (Table 1). A lower loss indicates that the state predictions of the proposed method are closer to the true droplet states. Furthermore, the loss and mIoU of the proposed method are more stable after convergence (Fig. 4e–k), indicating that it has learned and mastered effective semantic segmentation.

**Table 1:** Experimental data on the evaluation metrics of different semantic segmentation models

	mIoU	loss	mPrecision	mPA	mRecall
Proposed model	**89.73%**	**0.11**	**95.72%**	**93.30%**	**93.30%**
Traditional U-net	84.69%	0.14	92.96%	90.97%	90.37%
DeeplabV3+	82.53%	0.22	91.76%	89.80%	88.76%

The bolded values in Table 1 represent the superior results of the proposed model compared to the traditional U-Netand DeeplabV3+ on evaluation metrics, demonstrating that the proposed model achieved higher performance

The mean precision (mPrecision), mean pixel accuracy (mPA), and mean recall (mRecall) on the validation set are also calculated. The three metrics used to evaluate the performance of the model’s semantic segmentation results are category accuracy, pixel-level classification accuracy, and positive sample detection (Supplementary Information S2 Eqs. 1–5). The mPrecision is 95.72%, the mPA is 93.30%, and the mRecall is 93.30% (Table 1 and Supplementary Fig. S2). The three metrics of the proposed method are greater than 4%, 4%, and 5% for DeeplabV3+ and greater than 3%, 2%, and 3% for U-Net, with higher values indicating that the proposed method accurately recognizes objects in the image and generates segmentation results that closely match the actual labeled objects.

### Influence of the luminous environment

**Fig. 5:**
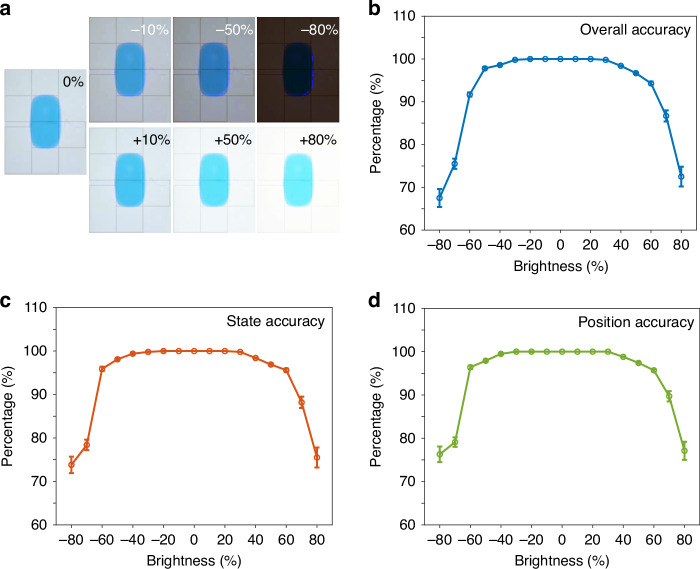
Recognition and segmentation results of 8 different real-time videos under different brightness levels. **a** Comparative images showing the original image and images with different brightness levels; **b** overall mean accuracy (±standard deviation, SD, *n* = 8) of different real-time videos at various brightness levels; **c** state mean accuracy (±SD) of different real-time videos at various brightness levels; **d** position mean accuracy (±SD) of different real-time videos at various brightness levels

To investigate the influence of the luminous environment in different DMF systems on the performance of the proposed method, a program based on OpenCV is used to perform linear transformations on the pixel values, multiplying the original pixel values by a coefficient factor, which can increase or decrease the overall brightness level of the images. Based on the brightness of the initial video, increasing or decreasing the coefficient factor by 10% each time gradually simulates the change in the luminous environment (Fig. 5a). The accuracy of the recognition of states, positions, and overall values under various luminous environments is also analyzed across the videos (Supplementary Information S2 Eqs. 6–8).

With a 0% and 50% increase or decrease in brightness, the overall accuracy decreases from 100% to 96.7% (Fig. 5b). Moreover, the mean accuracy for recognizing both states and positions consistently decreases from 100% to 97% (Fig. 5c, d); this shows that the proposed model can still maintain a high level of accuracy as long as changes in the luminous environment do not lead to blurring of droplet edges. With a 60% and 80% increase or decrease in brightness, the overall mean accuracy decreases from 91.7% to 67.5% (Fig. 5b). Similarly, the mean accuracy for state and position recognition decreases to 95.6% and 73.8% (Fig. 5c), respectively, and 95.7% and 76.3% (Fig. 5d), respectively. When the luminous environment variation approaches 80%, overexposure or underexposure occurs. The color of the droplet is changed, and its edges are no longer distinct, leading to a noticeable decrease in recognition accuracy. Therefore, especially in the luminous environment with a range of 0–50%, the proposed system is more accurate and robust.

### Influence of droplet color and shape

**Fig. 6:**
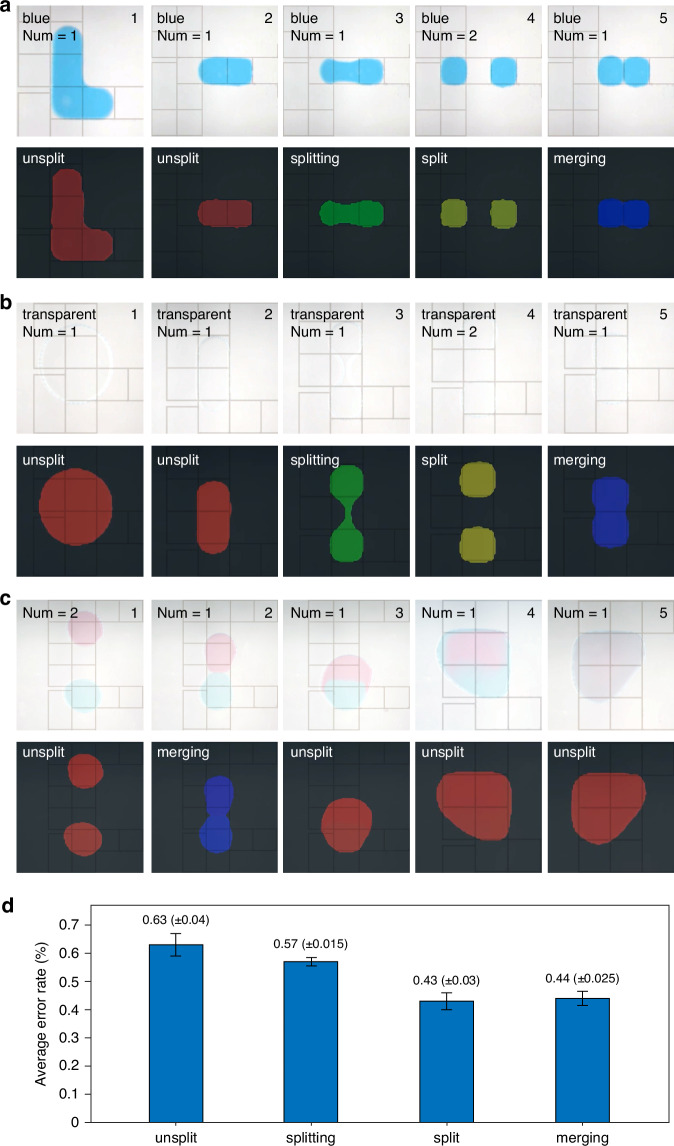
Recognition and segmentation results for droplets with different colors and shapes. **a** Results for recognition of blue droplets with different states (Num: number of droplets); **b** results for recognition of transparent droplets with different states; **c** results for a merging process of two differently colored droplets; **d** mean error rate (±standard deviation, SD, *n* = 5) for recognition in droplet manipulation with different states and colors (red, yellow, blue, black, and transparent)

In practice, the color and shape of the droplets in DMF could vary with different applications or control processes. Therefore, red, yellow, black, and blue droplets were introduced here to further test the proposed system (Fig. 6 and Supplementary Fig. S3a). The droplets are also controlled to form shapes, including L-shaped, rectangular, circular and triangular shapes (Fig. 6a1, b1, and Supplementary Fig. S3a). As a result, the recognition performance is not affected by the color or shape of the droplets. The state transitions of both droplets with different colors and transparent droplets are recognized (Fig. 6a2–a5, b2–b5, Supplementary Fig. S3b–e, Supplementary Video 1–2, and Supplementary Information S4).

A mixing process is also used to generate graduated color droplets by merging droplets with different colors. An example is used to demonstrate the process of moving a red droplet to merge with a blue droplet (Fig. 6c). Before merging, the droplets are recognized as unsplit (Fig. 6c1). Once the red droplet touches the blue droplet, it is recognized as merging (Fig. 6c2) to indicate the success of merging. After the droplets merge into one droplet, the droplet state returns to the unsplit state (Fig. 6c3). To promote the mixing of the compositions, the merged droplet is subsequently actuated to run in circles. Although graduated color was still observed inside the droplet during mixing, it was still correctly recognized as unsplit (Fig. 6c4, c5). The results support the generalizability and stability of the proposed system for droplet state recognition in a wide range of applications.

To evaluate the stability of the proposed system in continuous droplet state recognition, the error rate is used to estimate the percentage of the incorrect state output during a continuous droplet control process. The mean error rates for the 4 states of different colored droplets (red, yellow, blue, black, and transparent) are approximately 0.63%, 0.57%, 0.43% and 0.44%, respectively (Fig. 6d). The incorrect outputs are mainly induced by the transient deformation of the droplets, which would not greatly affect the further control process due to its low probability.

### AI-assisted multistate droplet control

To validate the applicability of the AI-assisted multistate feedback control system μDropAI, droplet manipulations, including moving, splitting, and dispensing on the DMF platform, are performed.

**Fig. 7:**
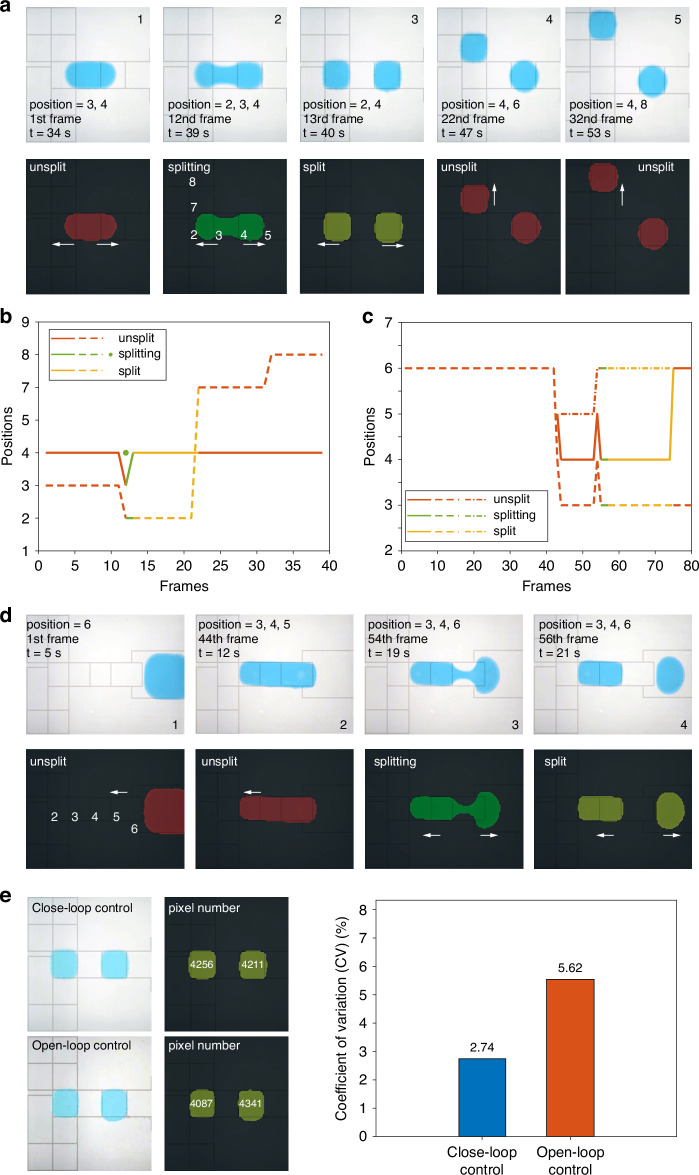
Processes and results of droplet movement, splitting, and dispensing. **a** The states and positions during droplet movement and splitting processes in different frames. **b** The state and position results in droplet movement and splitting processes. The horizontal axis represents the number of frames in the experiment, and the vertical axis represents the position of the droplets in the same frame. Multiple lines are utilized to indicate the droplet positions in each frame. **c** The state and position results for droplets dispensed from the liquid reservoir. **d** The states and positions of droplets dispensed from the liquid reservoir in different frames (electrode 6 is the reservoir electrode, electrode 5 is the dispensing electrode, and electrodes 2, 3, and 4 are the driving electrodes). **e** Comparison between the proposed closed-loop control method, which is based on the pixel count of the segmented droplet image, and the open-loop control method in terms of droplet splitting and the coefficient of variation in 70 experiments

Droplet movement and splitting in digital microfluidics are common biological and chemical experimental operations. A flow chart of automated feedback control involving droplet movement and splitting is shown (Supplementary Information S5, Supplementary Fig. S4a, c). In droplet movement manipulation, μDropAI recognizes the droplets and determines their locations on the platform. The activated electrodes subsequently drive the droplets to reach their destinations. When the droplet reaches the target electrode, μDropAI confirms that the droplet has successfully moved. (Fig. 7a4, a5, b and Supplementary Video 3). When the movement fails, the electrode must be reactivated to drive the droplet. Compared with existing image-based methods^27^, our system is more adaptable to a variety of environments, recognizing the edges of the droplets and distinguishing the states and positions of the droplets. In the droplet splitting process, the droplet undergoes a total of three state transitions, from the unsplit state of a signal droplet to the hourglass-shaped splitting state and finally to the split state of two smaller droplets (Fig. 7a1–a3, b and Supplementary Video 3). When two electrodes are activated sequentially, the droplet may be pulled to one side by the first activated electrode, leading to a failure in splitting. In such cases, the control system reverts the droplet to its initial state and resplits (Supplementary Video 4). Additionally, the system automatically merges the droplets and recognizes them. The proposed control system accurately detects the droplet state and controls the droplet operation according to the droplet state, extending beyond mere location-based manipulation determination.

Droplet dispensing is an operation that generates droplets from a reservoir. A flow chart of automated feedback control experiments for dispensing is shown in Supplementary Information S5 and Supplementary Fig. S5. When the initial position and state of the droplet are recognized, electrodes 3, 4, and 5 are activated to release the droplet from reservoir electrode 6. The proposed model recognizes whether the droplet has covered target electrodes 3 and 4 for dispensing (the pixel of the segmented droplet contains all the pixels of the target electrode) (Fig. 7d1, d2 and Supplementary Fig. S6b, c). The electrodes remain activated until the droplet covers the target electrodes. Reservoir electrode 6 is activated, and dispensing electrode 5 is closed to split the droplet, with the droplet being in the splitting state (Fig. 7d3). If the recognized droplet state transitions from splitting to split in a specified time (1 s), a small droplet has been dispensed (Fig. 7d4, c, Supplementary Fig. S6, and Supplementary Video 5); otherwise, a timeout event is activated to restart the process. After the droplet is dispensed, the pixel count of the segmented droplet image is obtained to evaluate the droplet volume. When the volume error of the dispensed droplet is less than 3%, the process is completed; otherwise, the system restarts the process. To create a droplet of one unit size, the driving electrode 3 is simply activated to dispense from the reservoir. The AI-assisted method improves the capability of automated droplet dispensing, making it highly applicable to a broader range of DMF applications.

The precision of droplet volume determination is crucial in various applications, such as drug discovery and quantitative analysis. Hence, a feedback closed-loop control method based on the pixel count of segmented droplet images is proposed to enable the precise splitting of droplets by monitoring droplet volumes and controlling electrodes (Supplementary Information S5 and Supplementary Fig. S4b). During the process of droplet splitting, the proposed semantic segmentation model is also utilized to calculate the pixel count of the split droplets to determine their respective volumes. If the volume error of the split droplets exceeds 3%, the droplet will be moved to another electrode to be re-split. According to the pixel count, the droplet volume is calculated (Supplementary Information S6 and Supplementary Fig. S7b). The coefficients of variation (CVs) are calculated for split droplets under the proposed closed-loop and open-loop control methods in 70 experiments. A larger CV indicates a greater volume error in the split droplets. The proposed method yields more evenly split droplets, with the CV value being limited to 2.74%, which is lower than the CV value of 5.62% (the CV value is basically consistent with that of traditional droplet dispensing^48^) (Fig. 7e and Supplementary Fig. S8).

The fundamental droplet operations shown above demonstrate the unprecedented advantages of the AI-assisted feedback control system for DMF. It can autonomously conduct experimental operations with various forms of reagents, such as protein-rich droplets, on DMF chips without human intervention. Moreover, it can be combined with reinforcement learning to increase the precision and automation of droplet manipulation by recognizing, tracking, and discriminating states as well as automating droplet control, thereby expanding the capabilities and applications of DMF systems.

## Conclusions

In summary, the AI-assisted digital microfluidic system μDropAI is presented in this paper. The system uses the U-Net based model for multistate droplet recognition and segmentation and employs a closed-loop feedback control strategy for real-time control during multistate droplet manipulations. The U-Net-based model is robust enough to recognize droplets at different brightness levels, while it can also segment droplets of different colors and shapes, different states, and even the merging of droplets of different colors. The AI-assisted DMF platform demonstrates the ability to automatically correct failed droplet manipulations by recognizing the droplet position and judging the manipulation state. Moreover, the AI-assisted platform also improves the uniformity of the split droplet volume. We envision that this AI-assisted feedback approach will be widely applied in other DMF platforms to achieve precise and automatic droplet manipulations in a wide range of automated DMF applications. Moreover, with the advancement of large-scale models such as ChatGPT, AI-assisted digital microfluidic systems can be integrated with ChatGPT to enable more intelligent manipulations.

Nonetheless, this study is a preliminary proof of concept, and we note several limitations of the current implementation. First, the model is too large to be integrated for computation on, for example, embedded devices, as this is more computationally time- and resource-intensive. To solve this problem, we plan to start with model reduction to ensure accurate recognition while reducing the number of parameters. In addition, the manipulation of multiple droplets is still difficult, and we intend to design algorithms to track the manipulation process of droplets and realize automatic feedback control of the manipulation of multiple droplets.

## Supplementary information

ESI

video 1

video 2

video 3

video 4

video 5
