# Supplementary material for: An artificial intelligence-assisted digital microfluidic system for multistate droplet control
Source: Microsyst Nanoeng. 2024 Sep 27;10:138. doi: 10.1038/s41378-024-00775-5 (PMC11427450; doi:10.1038/s41378-024-00775-5)
Supplement: Supplementary file 1 — ESI [file 41378_2024_775_MOESM1_ESM.docx]

**The electronic supplementary information includes:**

S1. Region growing algorithm;

S2. Equations of the state accuracy, position accuracy, overall accuracy, mean precision and mean pixel accuracy;

S3. The U-net model evaluation index of the pixels;

S4. Recognition and segmentation results under different colours, shapes, sizes, and states;

S5. Droplet splitting and moving and reservoir splitting droplet;

S6. Calculation of droplet volume;

Table S1 The structure of the semantic segmentation encoder-decoder model;

Table S2 Number of images of the dataset for 4 different droplet states;

Video 1-5. Experiment results of real-time recognition of the transparent droplet manipulations (video S1), experiment results of real-time recognition of the multi-state during droplet manipulations (video S2), and experiment results of automated feedback control of droplet splitting, moving, (video S3 and video S4) and droplet dispensing (video S5).

**S1. Region growing algorithm**

The region growing algorithm is an image segmentation method based on a specific detection criterion, whose basic idea is to form a series of regions by progressively aggregating pixels with similar properties in all directions from an initial point (or a pre-determined seed point) that possesses the ability to satisfy specific conditions. As shown in the Fig. S1, in each segmented region, by selecting a seed pixel as the starting point for growth, subsequently, neighboring pixels that are consistent or similar to that seed pixel under some a priori growth or similarity criterion are fused into the same region. The subsequently fused pixel will be considered as a new seed pixel, which in turn continues the above process. This iterative progression continues until there are no more mergeable pixels that satisfy the growth criteria, thus allowing the region to reach a steady state.


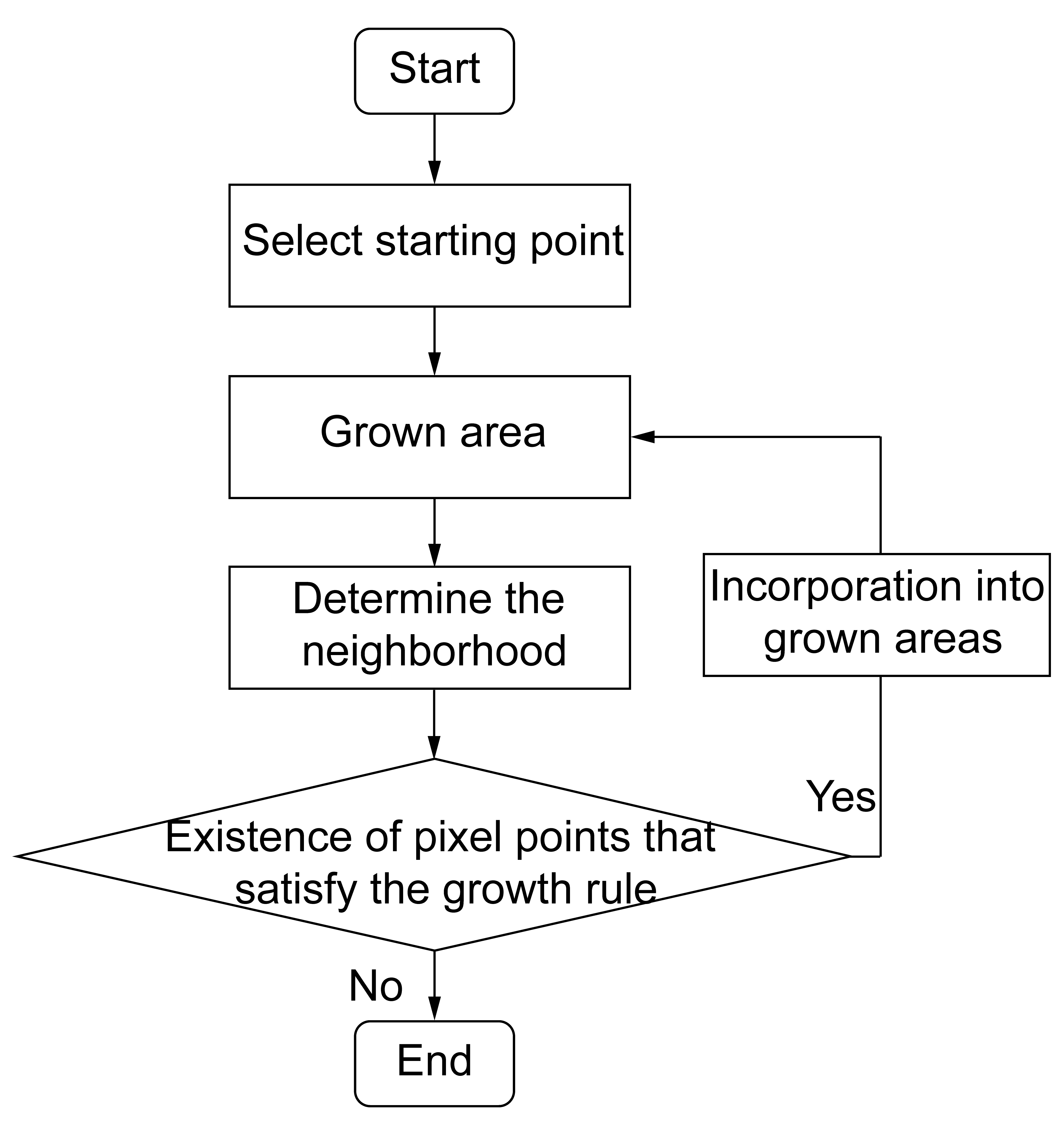


Fig. S1 Flow chart of region growing algorithm

**S2. Equations of the mean precision, mean pixel accuracy, mean recall, state accuracy, position accuracy, and overall accuracy**

The mean precision (mPrecision) refers to a metric that calculates the precision for each category in a multi-class scenario and takes the average of these values. The calculation equation is shown as Equ.1 and Equ.2.

$$mPrecision= \frac{precision_{1}+precision_{2}+\cdot\cdot\cdot+precision_{n}}{n}$$

$precision_{i}= \frac{TP_{i}}{TP_{i}+FP_{i}}$ (2)

Where, $n$ represents the total number of categories, $precision_{i}$represents the precision of the $i-th$ category. $TP$ represents the number of samples correctly predicted as positive by the model in all samples, while $FP$ represents the number of samples incorrectly predicted as positive by the model in all samples.

The mean pixel accuracy (mPA) refers to comparing each pixel predicted by the model with its corresponding ground truth label and calculating the ratio of correctly classified pixels to the total number of pixels, calculated as Equ.3.

$mPA= \frac{1}{n+1}\sum_{i=0}^{n} \frac{p_{ii}}{\sum_{j=0}^{n} p_{ij}}$ (3)

Where, $n$ represents the total number of categories, $p_{ii}$ represents the number of correct predictions,$p_{ij}$ represents predicting of $i$ as $j$, false negatives.

The Mean Recall (mRecall) is usually the average recall in a multi-category classification task. Used to measure the overall performance of the model in a multi-category classification problem, it considers the recall of each category and averages them. The calculation equation is shown as Equ.4 and Equ.5.

$mRecall= \frac{recall_{1}+recall_{2}+\cdot\cdot\cdot+recall_{n}}{n}$ (4)

$recall_{i}= \frac{PT_{i}}{PT_{i}+PF_{i}}$ (5)

Where, $n$ represents the total number of categories, $recall_{i}$represents the precision of the $i-th$ category. $PT$ represents the number of samples correctly predicted as positive by the model in all positive samples, while $PF$ represents the number of samples incorrectly predicted as positive by the model in all negative samples.

State accuracy is an algorithm's ability to recognize the current state of a droplet in each frame of a video. It is measured by comparing the recognized droplet states with the actual droplet states in the recorded video. The state accuracy is calculated by determining the percentage of correctly recognized droplet states over the total number of frames in the video, as shown in Equ.6.

$State accuracy= \frac{RSN}{FN} \times100\%$ (6)

Where, $RSN$represents the number of frames in which the droplet states are correctly recognized, and $FN$ represents the total number of frames in the real-time video.

Position accuracy refers to the algorithm's ability to recognize the precise position of a droplet in each frame of a video. It is measured by comparing the algorithm's recognized droplet positions with the actual droplet positions in the real-time video. The algorithm determines the droplet's position by identifying the location with the highest concentration of droplet pixels, and this location is considered as the droplet's position. Equ.7 represents the calculation of position accuracy, which is the percentage of correctly recognized droplet positions over the total number of frames in the video.

$Position accuracy= \frac{RPN}{FN} \times100\%$ (7)

Where, $RPN$ represents the number of frames in which the droplet positions are correctly recognized, and $FN$ represents the total number of frames in the real-time video.

Overall accuracy refers to the algorithm's ability to recognize both the state and position of droplets in each frame of a video. It is calculated by comparing the algorithm's recognized droplet states and positions with the actual droplet states and positions in the real-time video. The overall accuracy is determined by calculating the percentage of frames in which both the droplet state and position are correctly recognized, out of the total number of frames in the video, as shown in Equ.8.

$Overall accuracy= \frac{RSPN}{FN} \times100\%$ (8)

Where, $RSPN$ represents the number of frames in which both the droplet state and position are correctly recognized, and $FN$ represents the total number of frames in the real-time video.

**S3. The proposed model evaluation index of the pixels**





Fig. S2 The comparison evaluation index on the pixels of the DeeplabV3+, traditional U-net, and the proposed. (a) The mPrecision of the DeeplabV3+; (b) The mPrecision of the traditional U-net; (c) The mPrecision of the proposed; (d) The mPA of the DeeplabV3+; (e) The mPA of the traditional U-net; (f) The mPA of the proposed; (g) The mRecall of the DeeplabV3+; (h) The mRecall of the traditional U-net; (j) The mRecall of the proposed.

**S4. Recognition and segmentation results under different colors and shapes**

As shown in Fig. S3, we recognize droplets of different colors and shapes. Fig. S3a shows that the algorithm distinguishes the droplets from the background and accurately recognizes that the droplets are in the "unsplit" state, regardless of whether the droplets are black, yellow, blue, or even transparent, and regardless of whether the droplets are in the shape of a straight line, a square, an l-shape, or a triangle. We also discuss the change in state of droplets of different colors as they split. When a droplet is hourglass-shaped, it is recognized as a "splitting" state. When a large droplet splits into two small droplets, the splitting process is complete and the state changes to "split". When two splitting droplets merge, the state is recognized as "merging" (Fig. S3b-d). The state changes all match the fluidic changes of the droplets during the actual operation, which proves that our system has a strong generalization ability to recognize the instantaneous operational state changes of droplets with different colors and shapes.





Fig. S3 Recognition and segmentation results under different colors, shapes. (a) The recognition results of different colored and shaped droplets; (b) The recognition states of red droplets; (c) The recognition states of yellow droplets; (d) The recognition states of black droplets; (e) The error rate of different colored droplets (the error rate is the error caused by the droplets being recognized as other states while remaining in the continuous and same state).

**S5. Droplet splitting, moving, and droplet dispensing**

A flow chart of automated feedback control involving droplet splitting is performed in the Fig. S4a. Droplet splitting: First, we will activate the electrode where the droplet is located, then open the electrodes on both sides of the droplet for stretching, and then close the middle electrode for splitting. The current state of the droplet is recognized by the semantic segmentation model to determine whether the splitting process has been completed in a configured time (1s). When the droplet state is "split", the splitting process is successfully completed. Otherwise, a timeout event will be activated to restart the process.

A flowchart of closed-loop control method for droplet splitting based on pixel values is shown in Fig. S4b. After the droplet splitting is completed, the pixel count of segmented droplet image is counted to evaluate the droplet volume. When the volume error of the split droplet is less than 3%, the process is completed; otherwise, the system will merge the droplets, move to another electrode, and split again.

A flow chart of automated feedback control involving droplet moving is performed in the Fig. S4c. Droplet movement: First, we will give the next target position according to the current position of the droplet. When the droplet moves in a configured time (1s), it can determine whether the droplet reaches the target position according to the droplet position recognized by the semantic segmentation model. Otherwise, a timeout event will be activated to restart the process.


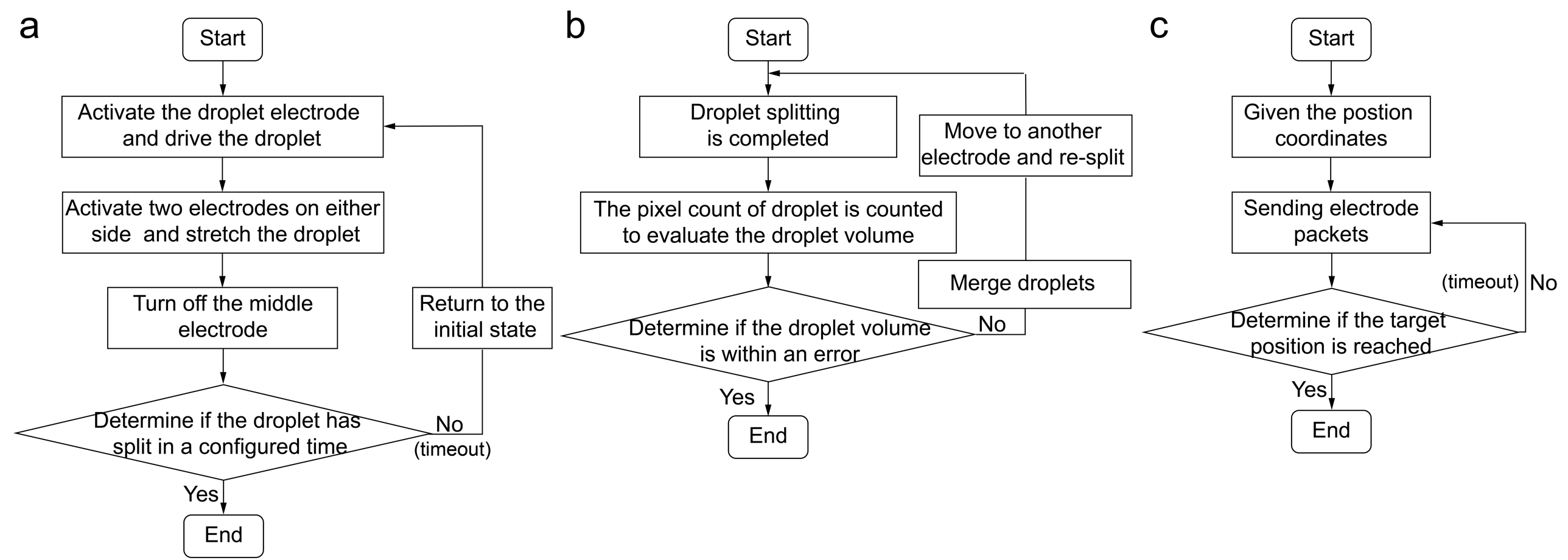


Fig. S4 (a) Flow chart for automated feedback control of droplet splitting; (b) Flow chart of closed-loop control method for droplet splitting based on pixel values; (c) Flowchart of automated feedback control of droplet moving.

A flow chart of automated feedback control experiments for dispensing is performed in Fig. S5. Droplet dispensing: with recognizing the initial position and state of the droplet, electrodes are activated to release the droplet from the liquid reservoir. The proposed model will recognize whether the droplet has covered the target electrodes for dispensing (the pixel of segmented droplet contains all the pixel of the target electrode). The electrodes will keep activated until the droplet covers the target electrodes. Then the system will keep the reservoir electrode activated and close the middle electrode to split the droplet. If the droplet state is recognized as "split" in a configured time (1s), the system will proceed to the next step. Otherwise, a timeout event will be activated to restart the process. After the droplet dispensing, the pixel count of segmented droplet image is counted to evaluate the droplet volume. When the volume error of the dispensed droplet is less than 3%, the process is completed; Otherwise, the system will restart the process.


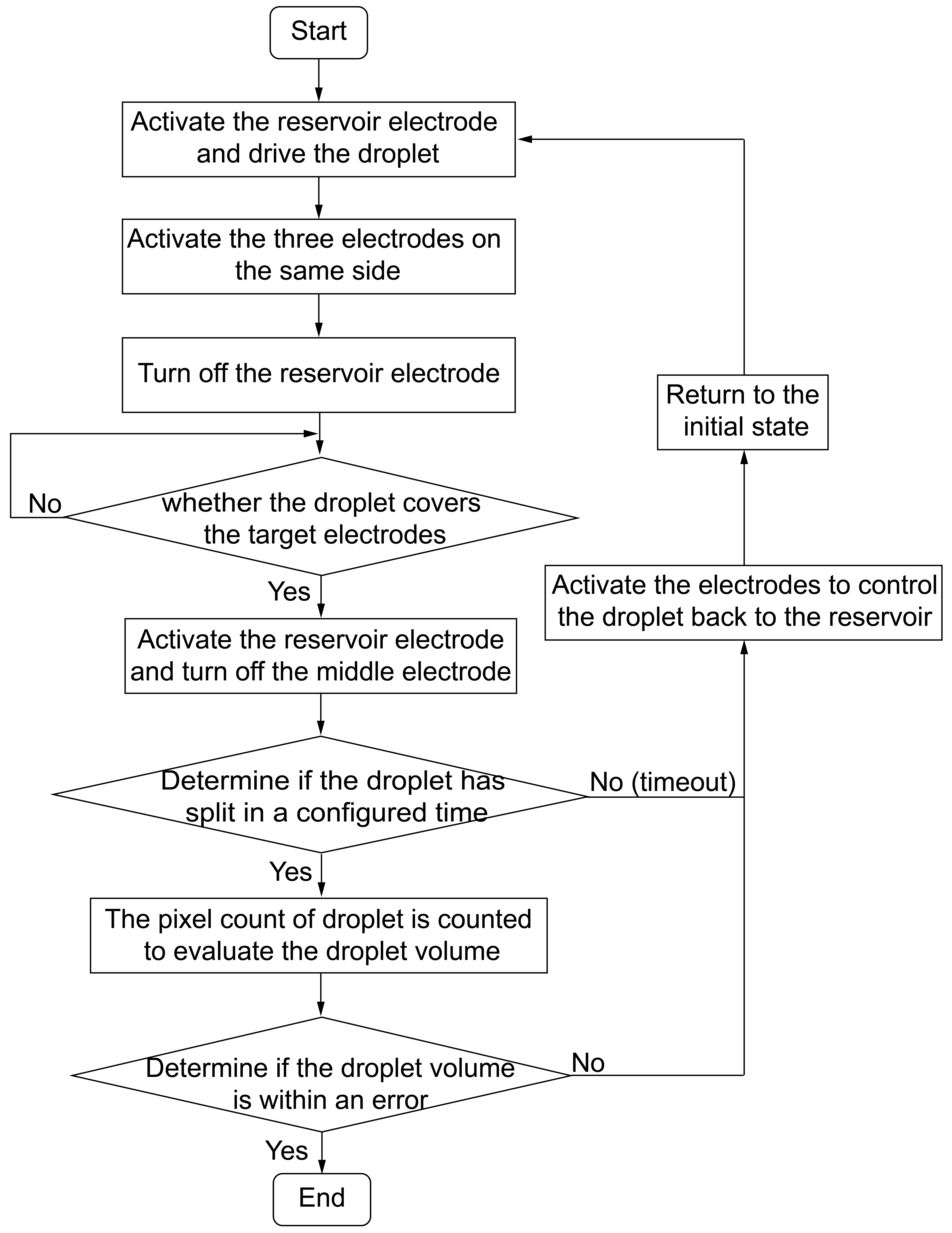


Fig. S5 Flow chart of automated feedback control of droplet dispensing.


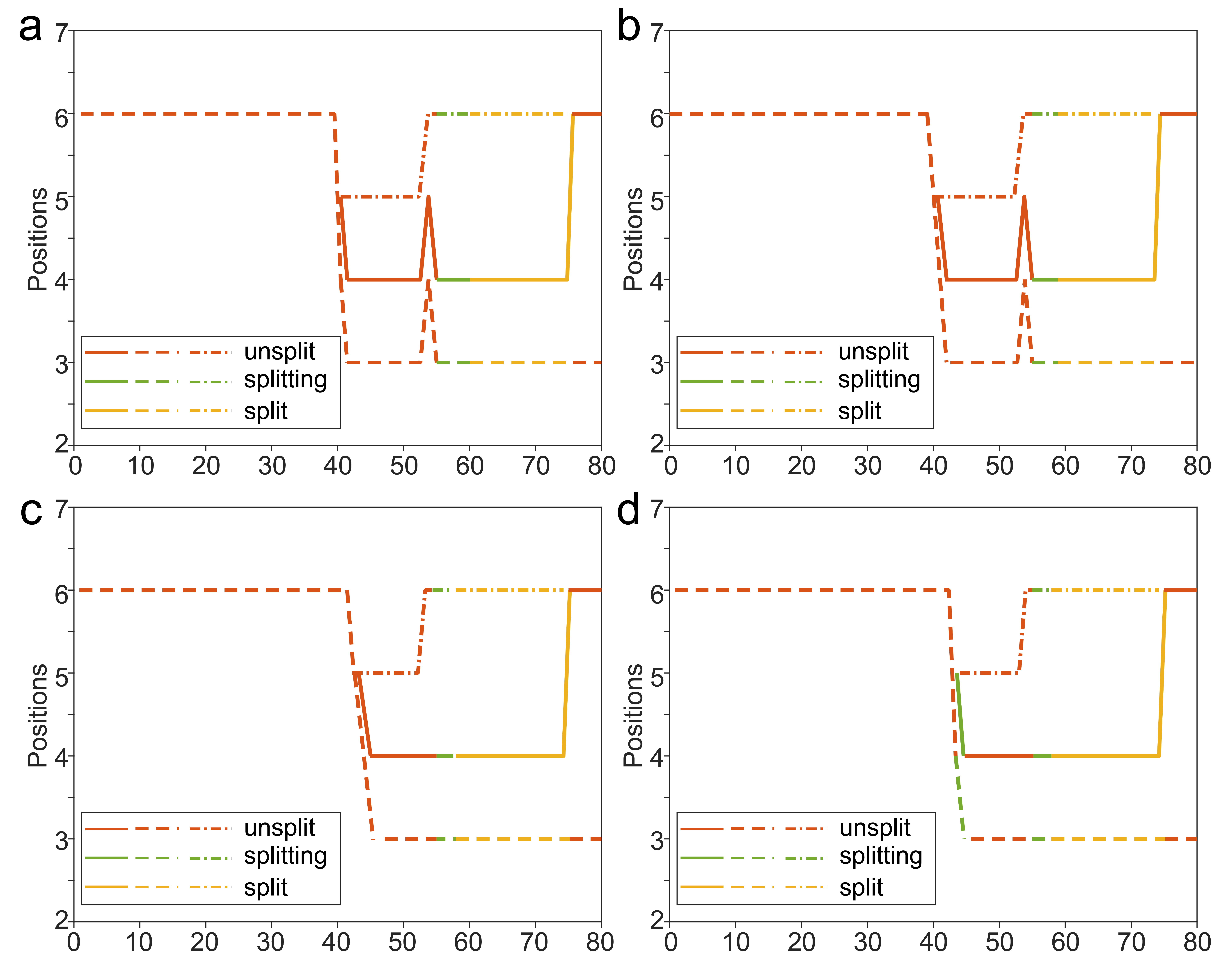


Fig. S6 The state and position results in droplet dispensing from liquid reservoir.

**S6. Calculation of droplet volume**

**Linear model**: since the channel height between the top and bottom plates is fixed, the droplet area determines the droplet volume. The droplet projection area can be recognized as the droplet pixel count. As a result, the droplet volume $V$ can be related to droplet pixel count $P$ as:

$V=mP+n$ (9)

where, $m$ is the coefficient, $n$ is the constant.

The droplet projection area $S$ on the plate can then be related to droplet pixel count $P$ as:

$S=o\times P$ (10)

where, $o$ is the coefficient.

**Nonlinear model**: As shown in the Fig. S7a, the droplet volume $V$ can be related to the droplet contact radius $a$ as:

$V=\pi a^{2}H+2\pi r^{2}\left[af_{1}(\theta)+\frac{H}{2}f_{2}(\theta)\right]$ (11)

where:

$f_{1}(\theta)=\theta-\frac{\pi}{2}+\frac{\sin(2\theta-\pi)}{2}+2\sin(\theta)\cos(\theta)$ (12)

and

$f_{2}(\theta)=\tan(\theta)\left[\theta-\frac{\pi}{2}+\frac{\sin(2\theta-\pi)}{2}+2\cos(\theta)\right]-\frac{1}{3}\cos^{2}(\theta)+(1-\sin(\theta))^{2}$ (13)

The radius of the interface $r$ in terms of the droplet contact angle $\theta$ and the channel height $H$ can be written as:

$r=\frac{-H}{2\cos\theta}$ (14)

The droplet projection area $S$ on the plate can then be related to droplet projection radius $R$ as:

$S=\pi R^{2}$ (15)

Also, based on the contact angle, the relationship between the droplet projection area $S$ and the contact radius $a$ can be written as:

$a=\sqrt{\frac{S}{\pi}}-(1-\sin\theta)r$ (16)

Therefore, the droplet volume $V$ can be related to droplet projection area $S$ as:

$V=HS+2\sqrt{\pi}r(r^{2}f_{1}(\theta)-(1-\sin\theta)H)\sqrt{S}+\pi r^{2}H(f_{2}(\theta)+(1-\sin\theta)^{2})-2\pi r^{2}(1-\sin\theta)f_{1}(\theta))$ (17)

In Equ.17, the channel height between the double plates and the contact angle of the droplet are fixed, the $\pi r^{2}(Hf_{2}(\theta)-2bf_{1}(\theta))$ is the constant. Therefore, the droplet volume $V$ is determined by the area $S$ and $\sqrt{S}$.

The droplet projection area can be recognized as the droplet pixel count, the droplet volume $V$ can be related to droplet pixel count $P$ as:

$V=jP+k\sqrt{P}+l$ (18)

where, $j$ and $k$ are the coefficient, $l$is the constant.

In our experiments, we measure pixel count of different droplet volumes (2μl, 4μl, 6μl, 8μl, 10μl) and repeat the experiments 60 times for each volume. The parameters $H$ and $\theta$ are set to 0.5 mm and 110 degrees, respectively. As illustrated in Fig. S7b, the results suggests that both the linear and nonlinear can be used to evaluate the droplet volume for droplets with volume ranging from 2 μL to 10μL (the range of droplet volume commonly used in our DMF system).


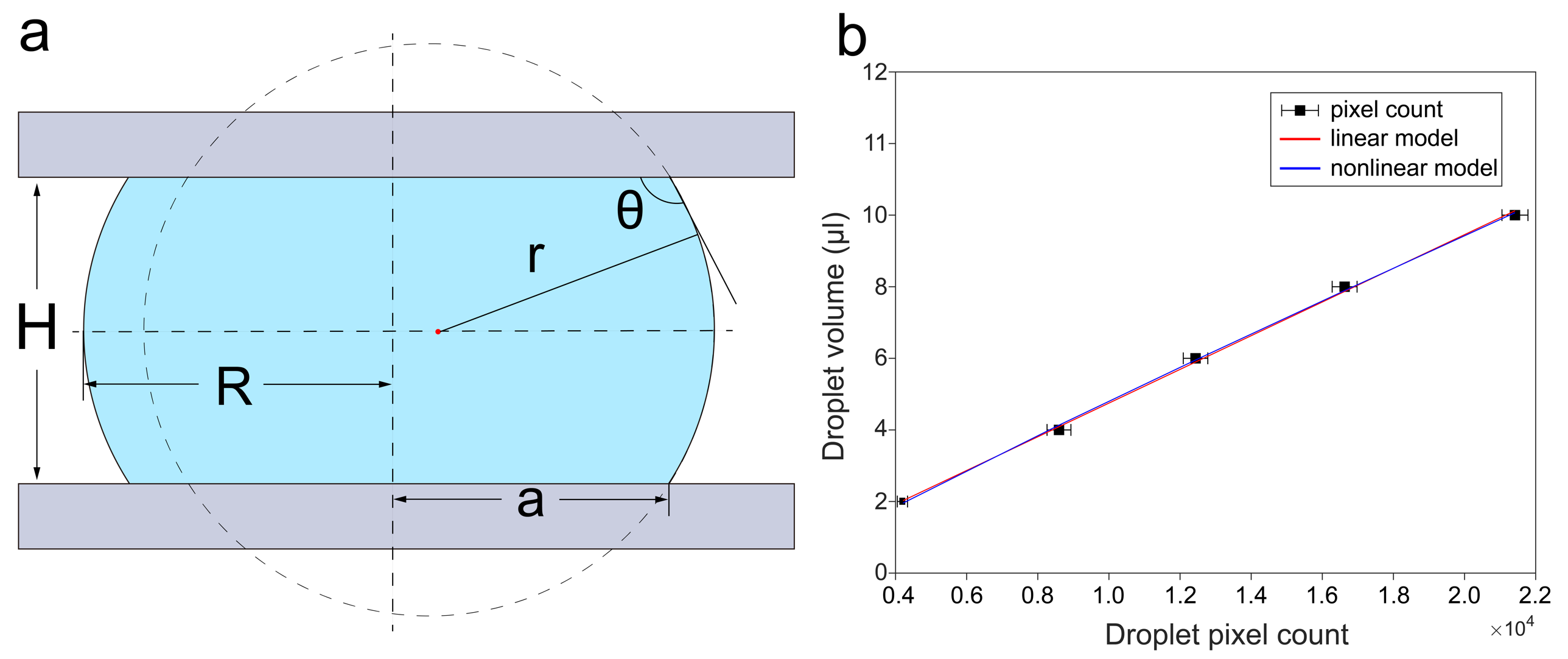


Fig. S7 (a) The modeling parameters are shown; (b) The linear and nonlinear relationship between droplet pixel count and droplet volume (n=60; the linear model (y = 0.00048 * x + 0.0363), correlation coefficient R^2^ = 0.9987; the nonlinear model (y = 0.00041 * x + 0.0164 * x^0.5^ - 0.7766), correlation coefficient R^2^ = 0.9990).


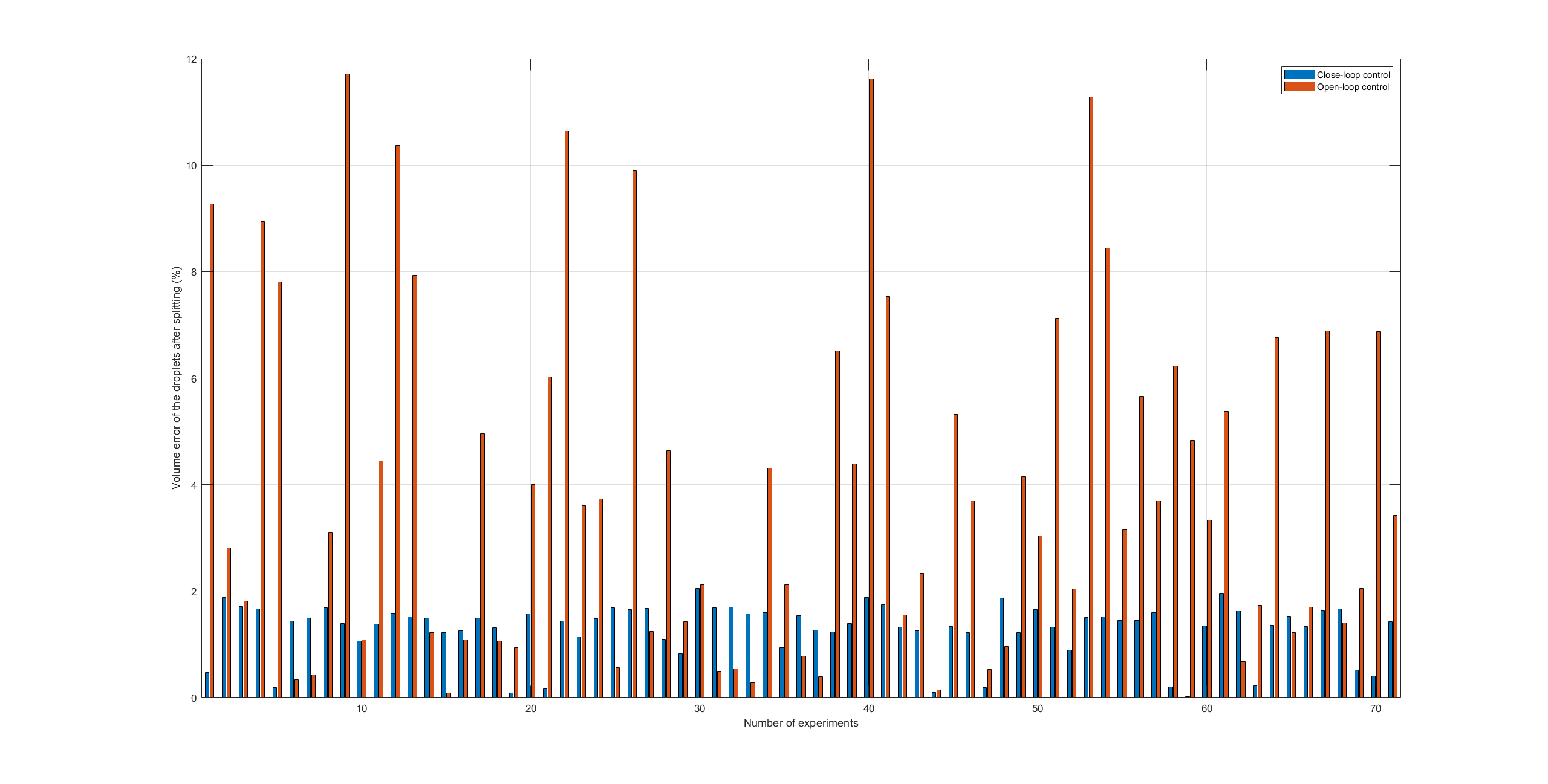


Fig. S8 The difference between the volume after droplet splitting under two control methods (the proposed close-loop and open-loop) and the average volume (2μL) in 70 experiments.

The proposed system can capture the entire region of the DMF chip, while simultaneously recognizing and processing the operations involving multiple droplets on the chip, and it is applicable to DMFs with various electrode layout configurations. The photograph of the DMF chip, the whole field of view captured by camera, as well as the segmented image is shown in Fig. S9.


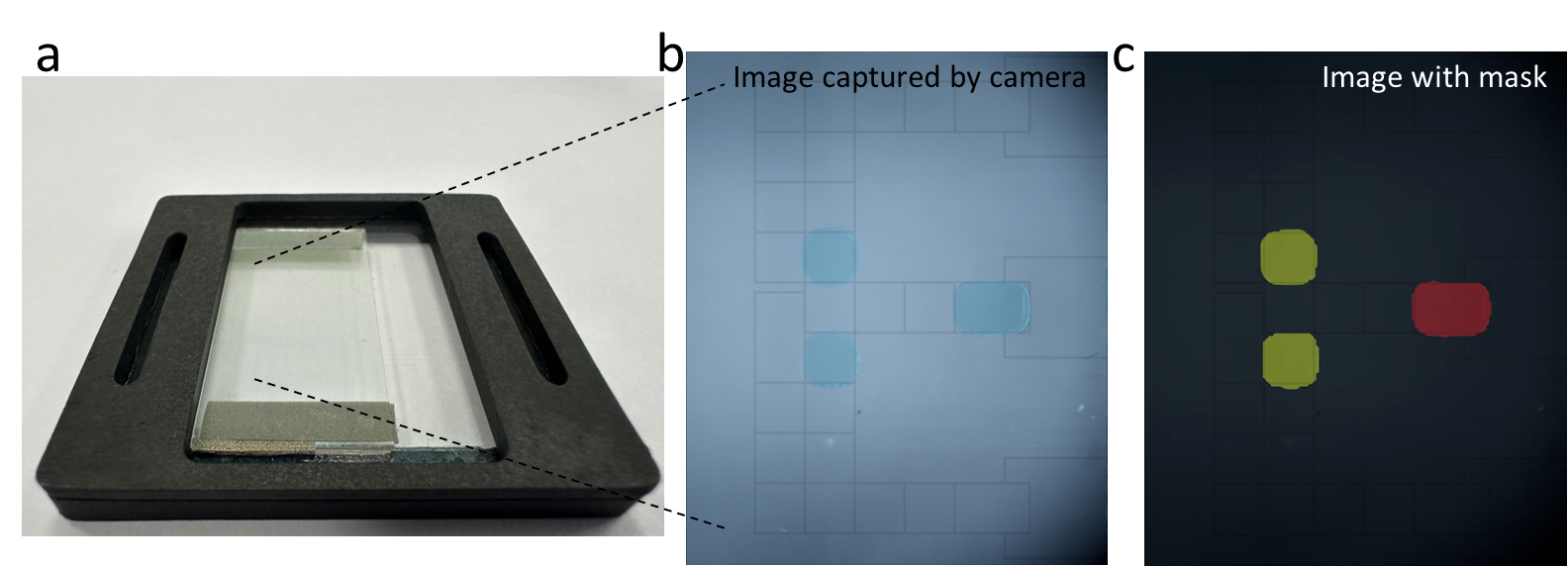


Fig. S9 Image of a physical DMF chip and droplet imaging on the chip captured by a camera. (a) Photograph of the DMF chip; (b) The image captured by camera; (c) The segmented image (red: "unsplit" state, yellow: "split" state).

Table S1 The structure of the semantic segmentation encoder-decoder model


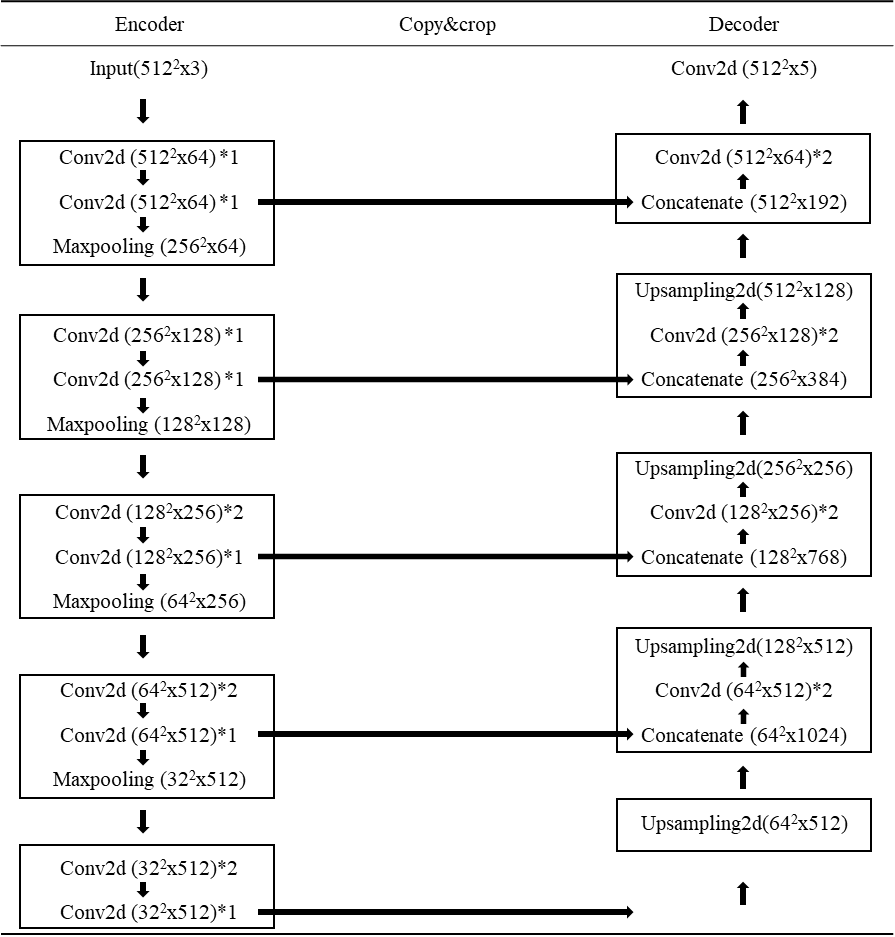


Table S2 Number of images of the dataset for 4 different droplet states

| Labels | Number |
| --- | --- |
| unsplit | 1416 |
| splitting | 1238 |
| split | 901 |
| merging | 824 |
